# Patient reported outcome measures in a large cohort of patients with type 1 Gaucher disease

**DOI:** 10.1186/s13023-020-01544-z

**Published:** 2020-10-13

**Authors:** Tama Dinur, Majdolen Istaiti, Dafna Frydman, Michal Becker-Cohen, Jeff Szer, Ari Zimran, Shoshana Revel-Vilk

**Affiliations:** 1 Gaucher Unit, Shaare Zedek Medical Centre, P.O. Box 3235, 91031 Jerusalem, Israel; 2 Faculty of Medicine, The Hebrew University of Jerusalem, Jerusalem, Israel; 3 Peter MacCallum Cancer Centre, Royal Melbourne Hospital, Melbourne, Australia; 4 Department of Medicine, University of Melbourne, Melbourne, Australia

**Keywords:** Gaucher disease, Patients reported outcome, Mobile survey, Adult patients, Quality of life

## Abstract

**Background:**

It is now acknowledged that the input of patients in health outcome assessment is vital to understanding the impact of diseases and interventions for those diseases. This study is the first report of patient-reported outcome measures (PROM) in a large cohort of patients with type 1 Gaucher disease (GD1) enabling us to study predictors of the reported outcomes.

**Method:**

The PROM was sent via a mobile phone survey to 405 adult patients with GD1. Demographics, clinical data, and treatment status were extracted from clinic charts. Age, sex, severity score index (SSI) at presentation and treatment status were used as variables to assess outcomes.

**Results:**

A total of 192 patients with GD1 (111 females) responded (47.4% response rate), of whom 124 (64.5%) had received GD1-specific therapy. Around 40% of patients reported that GD had restricted their education/job and fun activities and were concerned about being emotional and financial burdens on others. Concerns regarding the risk of bone disease and Parkinson disease were also high (60%). The severity of GD1 (reflected by the need for GD1-specific therapy and a high SSI) was associated with GD1-related restrictions and concerns, fatigue, physical weakness, bone pain, and worry regarding the future.

**Conclusions:**

The use of GD1 specific PROM highlights personal problems that are not captured by traditional outcome parameters and that need to be addressed to improve health-related quality of life. Validated PROM should be included among the outcome measures in clinical practice and future prospective studies for patients with chronic and rare diseases.

## Introduction

Patient-reported outcome measures (PROM) were developed as a method to ascertain and quantitate patients' views of their symptoms, their functional status, and their health-related quality of life (HRQoL) [1]. Patients' reports are the best available and the most reliable and valid method for obtaining information on unobservable events such as pain and fatigue. The PROM provides unique information on a patient's perception of both the disease and the management of the disease that could not be collected in any other way [2, 3].

Gaucher disease (GD), one of the two most common inherited lysosomal storage disorders, is known for its phenotypic heterogeneity [4, 5]. Patients with type 1 GD (GD1) may present with significant clinical features such as hepatosplenomegaly, thrombocytopenia, anemia, fatigue, and bone disease, whereas other patients with GD1 may be very mildly affected or even asymptomatic [6]. With the introduction of disease-specific therapy for patients with GD1 in the form of enzyme replacement therapy (ERT) and substrate reduction therapy (SRT), the wellbeing of patients with GD1 has largely improved, but not without significant cost to the patient and society [7, 8]. Using PROM to understand the health status and treatment effects of patients with GD1 in the current era is an important step towards improving patient care [9].

In the past, PROM in patients with GD used non-disease specific tools, such as the Short-Form-36 and Brief Pain InventoryEuroQoL-5D, with limited responsiveness of PROM to small differences between groups of disease phenotype [10]. Recently, a GD1-specific PROM (GD1-PROM) was developed with input from patients, which included 15 questions; six Point Verbal Response Scale regarding the last month and nine Visual Analogue Scales (VAS) from 0 to 10 regarding the previous week. This study is the first to report PROM and predictors for PROM in a large cohort of patients with GD1 using a mobile version of GD1-PROM.

## Methods

The GD1-PROM was developed by Shire/Takeda based on the input of adult patients with GD and of worldwide physicians managing patients with GD. Content validity of the GD1-PROM was completed, including rounds of combined concept elicitation and concept debriefing interviews. The GD1-PROM was developed specifically for patients with GD1 and was validated in patients with GD1. A certified Hebrew-translated version of GD1-PROM was provided to us by the developers (Shire/Takeda) [11]. A Google Docs-based survey, constructed from the items of the GD1-PROM, was sent via mobile SMS notice to 405 adult patients (age ≥ 18 years) with GD1 who had visited the Gaucher Unit at Shaare Zedek Medical Center (SZMC) at least once in the previous 2 years and who had a mobile device. The study design was approved by the SZMC IRB. An IRB waiver was received for signing a full-length informed consent. In the SMS notice, there was an explanation regarding the PROM, and if interested in proceeding, patients were prompted to insert their ID number before answering the PROM. Only responses with IDs were collected for this report. The IRB approved the publication of the anonymous data.

Demographics, clinical data, and treatment status were extracted from clinic charts. Based on the definition of "generation" we categorized five age groups (18–25, 26–43, 44–55, 56–73, 74 and older) [12]. Untreated patients were defined as patients who had never received any specific GD- treatment (ERT or SRT). The severity scoring index (SSI) at the time of presentation was calculated [13]. In our unit, levels of lyso-Gb1 are done routinely at each clinic visit. The levels drawn in the clinic visit closest to the date of answering the PROM were collected from the clinic charts.

### Statistical analysis

Results are presented as median and range. T-test and Mann–Whitney were used to compare normally distributed and non-parametric continuous data in independent samples, respectively. A Chi-square test was used to compare categorical data. Proportional odds logistics regression was used to analyze the Likert scale data. The variables included in the analysis were age, gender, SSI at the time of presentation, recent lyso-Gb1 levels and treatment status. Since multi-comparisons were done, results were considered to be statistically significant when two-tailed p-values were ≤ 0.01.

## Results

A total of 192 (47.4%) active adult patients with GD1 responded to the mobile phone survey (Table 1). Two-thirds of the patients were homozygous for the c.1226A>G (N370S) mutation. A third of patients followed in the Gaucher unit never received GD specific therapy. Those untreated patients were mainly homozygous for the c.1226A>G (N370S) mutation, diagnosed at an older age, and had a lower SSI (Table 1). The age, sex, SSI, and homozygosity to the c.1226A>G (N370S) mutation of those that responded (study cohort) were similar to the entire SZMC database.

**Table 1:** Characterization of adults' patients with Gaucher disease type 1 in our unit and in the study cohort

	SZMC unit^b^	Study cohort			
		Total	Untreated	Treated	p value*
Number	–	192	68	124	
Age, years^a^	Untreated 45 (22–83) Treated 49 (19–91)	48 (20–91)	47 (20–76)	49.5 (22–91)	NS
Female	Untreated 61% Treated 54%	111 (57.8%)	43 (62.3%)	68 (54.8%)	NS
Severity score index^a^	Untreated 2 (0–13) Treated 7 (1–25)	5 (0–25)	2 (0–13)	7 (2–25)	< 0.0001
Homozygosity to the c.1226A>G (N370S) mutation	Untreated 80% Treated 62%	133 (69.3%)	57 (83.8%)	75 (60.5%)	0.001

^*^Comparing treated and untreated patients in study cohort

^a^Median(range)

^b^Data were extracted from the SZMC database

The responses to the Likert scale questions regarding the previous month are presented in Table 2. The responses to six questions were significantly different between treated and untreated patients (Table 2). Almost all of the untreated patients reported that the GD did not restrict their education/job and fun activities, whereas 25%–30% of treated patients reported that the GD did restrict their education/job and fun activities a little or some of the time. Untreated patients were significantly less concerned about being at risk for bone disease and of GD-related medical issues compared to treated patients.

**Table 2:** Responses on the Likert scale questions over the past month in patients receiving Gaucher-disease specific therapy (Yes) and in untreated patients (No)

Questions	None of the time		A little of the time		Some of the time		Most of the time		All of the time		p value*
Treatment status	Yes	No	Yes	No	Yes	No	Yes	No	Yes	No	
1. My GD has restricted my education/job	80 (67%)	64 (94%)	17 (14%)	4 (6%)	19 (16%)	0	3 (2.5%)	0	1 (0.5%)	0	*0.002*
2. My GD has restricted fun activities with friends	94 (76%)	66 (97%)	14 (11%)	2 (3%)	12 (10%)	0	2 (1.5%)	0	1 (0.5%)	0	*0.002*
3. My GD has restricted my ability to have intimate relationships with my spouse/partner	94 (90%)	61 (100%)	6 (6.5%)	0	4 (2%)	0	0	0	0	0	*0.001*
4. My GD has restricted my ability to take part in hobbies and leisure activities	88 (71%)	61 (90%)	17 (14%)	4 (6%)	12 (10%)	3 (4%)	4 (3%)	0	2 (2%)	0	0.013
5. I have been concerned that I am an emotional burden to others because of my GD	94 (77%)	64 (95.5%)	13 (11%)	3 (4.5%)	10 (7.5%)	0	3 (2.5%)	0	2 (2%)	0	0.016
6. Because of my GD, I am concerned I will be at risk of bone disease	35 (28.5%)	38 (56%)	31 (25%)	16 (23.5%)	39 (32%)	13 (19%)	10 (8%)	0	8 (6.5%)	1 (1.5%)	*0.007*
7. Because of my GD, I am concerned I will be at risk of cancers	78 (65%)	49 (73%)	19 (16%)	10 (15%)	21 (17.5%)	7 (10.5%)	0	0	2 (1.5%)	1 (1.5%)	0.3
8. Because of my GD, I am concerned I will be at risk of Parkinson	43 (36%)	40 (59%)	25 (21%)	15 (22%)	46 (38%)	11 (16%)	3 (2.5%)	1 (1.5%)	3 (2.5%)	1 (1.5%)	0.1
9. Because of my GD, I am concerned I will be a financial burden	62 (50.5%)	48 (72%)	28 (23%)	11 (16%)	23 (19%)	8 (12%)	3 (2.5%)	0	6 (5%)	0	0.06
10. I am concerned I will not get the best therapy because of budget issues	72 (60%)	36 (54.5%)	23 (19.5%)	19 (29%)	16 (13%)	7 (10.5%)	6 (5%)	3 (4.5%)	3 (2.5%)	1 (1.5%)	0.9
11. I am concerned I may not have an expert physician for advice in the future	55 (45%)	32 (47%)	28 (23%)	19 (27.5%)	30 (24%)	12 (18%)	6 (5%)	4 (6%)	4 (3%)	1 (1.5%)	0.3
12. My non-Gaucher problems are more concerning than the Gaucher concerns	9 (7.5%)	5 (8%)	12 (10%)	7 (11%)	46 (38.5%)	19 (29%)	39 (33%)	17 (26%)	13 (11%)	17 (26%)	0.05

	Strongly agree		Agree		Neither/nor agree		Disagree		Strongly disagree		
13. My health in general has improved because of my Gaucher-specific medication	23 (25.5%)	NA	23 (25.5%)	NA	16 (18%)	NA	28 (31%)	NA	0	NA	NA
14. Over the past month, all of my medical concerns have been Gaucher-related	5 (4%)	1 (1.5%)	11 (9%)	2 (3%)	15 (12.5%)	2 (3%)	53 (43.5%)	19 (28%)	38 (31%)	44 (64.5%)	*0.002*
15. My current medication has treated my Gaucher-specific concerns	41 (34%)	3 (5%)	31 (26%)	0	11 (9%)	3 (5%)	18 (15%)	10 (16%)	19 (16%)	46 (74%)	*NA*

*GD* Gaucher disease, *NA* not applicable

^*^Proportional Odds logistic regression adjusted for age, gender and severity score index

**Fig. 1:**
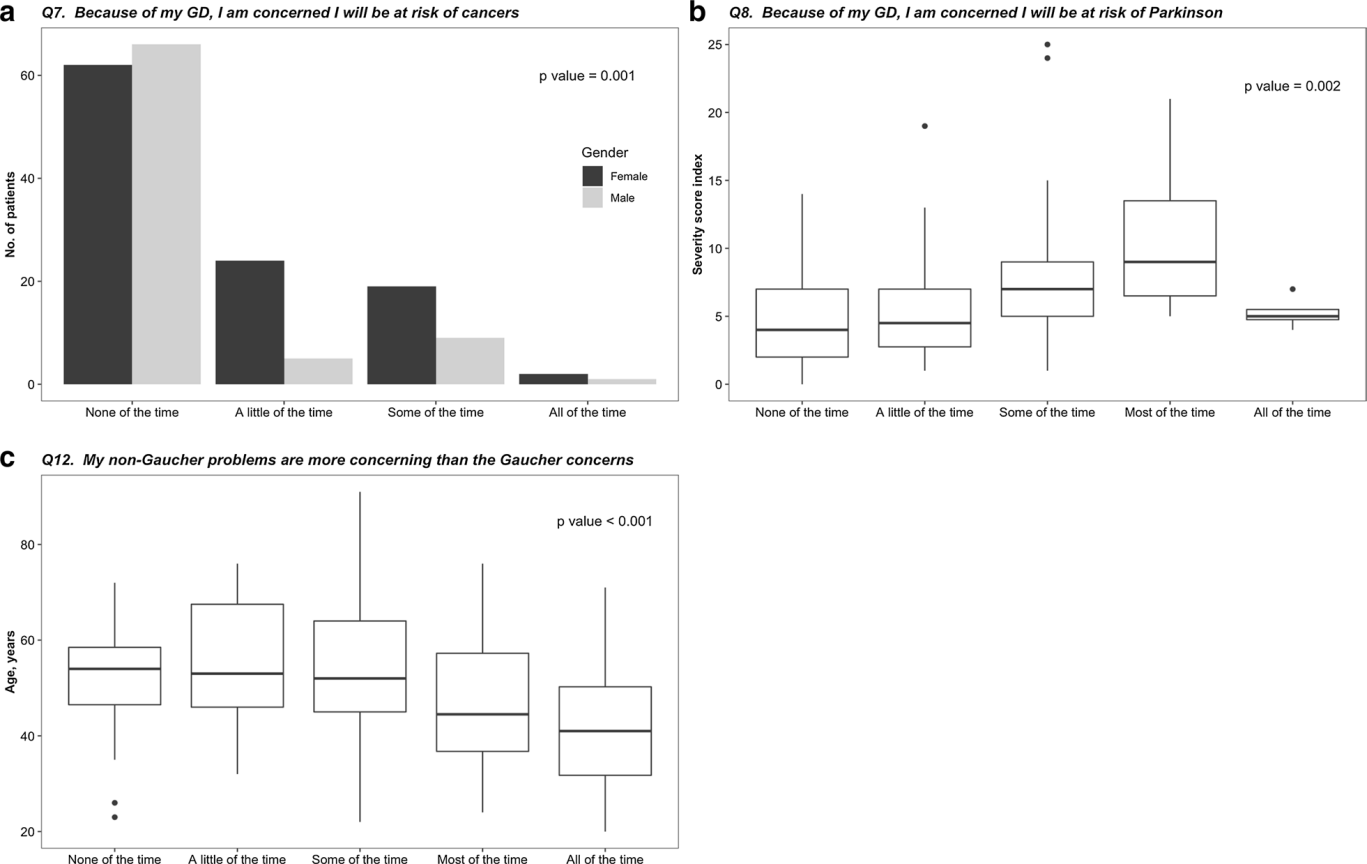
Significant associations between the responses on the Likert scale questions and patients' characteristics, i.e., sex, severity score index (SSI) at presentation and age. **a** The responses of women and men regarding the concern of the risk of cancers, **b** The distribution of the SSI of patients responding to their concern for the risk of developing Parkinson disease, **c** The distribution of the age of patients responding to the question on non-Gaucher problems compared to the Gaucher concerns

**Fig. 2:**
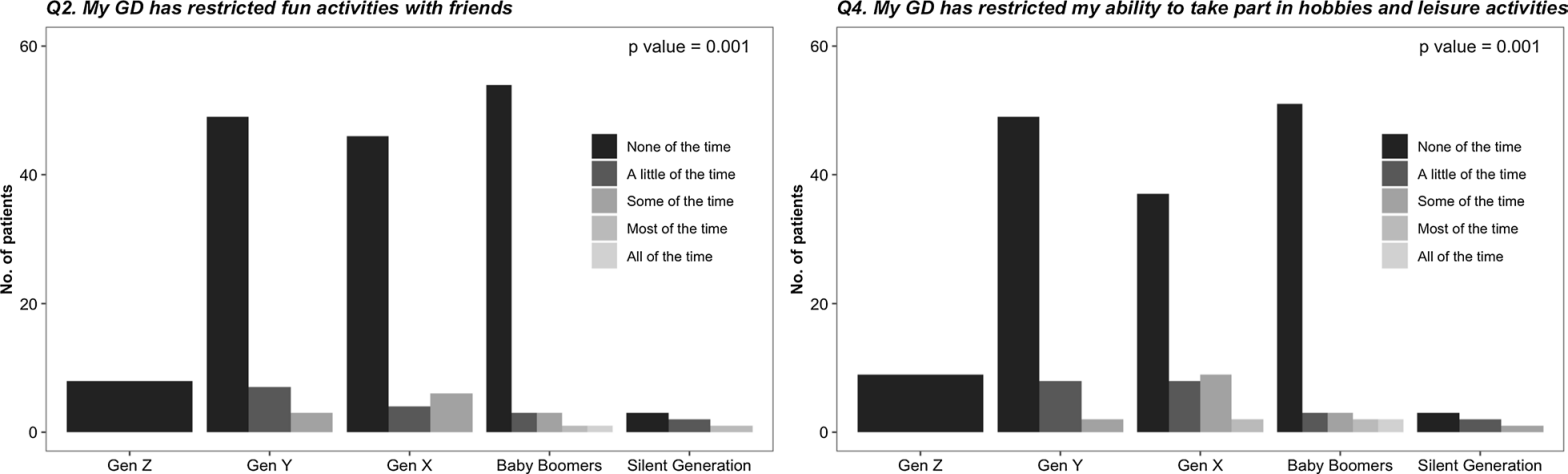
Significant associations between the responses on the Likert scale questions and the five age groups defined by "generation" (ref 12). Gen Z: 18–25 years, Gen Y: 26–43 years, Gen X: 44–55 years, Baby Boomers: 56–73 years, Traditionalists/ Silent Generation: 74 years and older

In the multivariate ordinal model, women were significantly more concerned about the risk of developing cancer (Fig. 1a, p = 0.001), higher SSI was associated with concern for the risk of developing Parkinson disease (Fig. 1b, p = 0.002) and younger age was associated with less concern from their GD (Fig. 1c, p < 0.001). Age categories per generation were associated with restrictions in fun activities with friends and the ability to take part in hobbies and leisure activities (Fig. 2). Sex and lyso-Gb1 levels were not associated with the responses on the Likert scale questions.

**Fig. 3:**
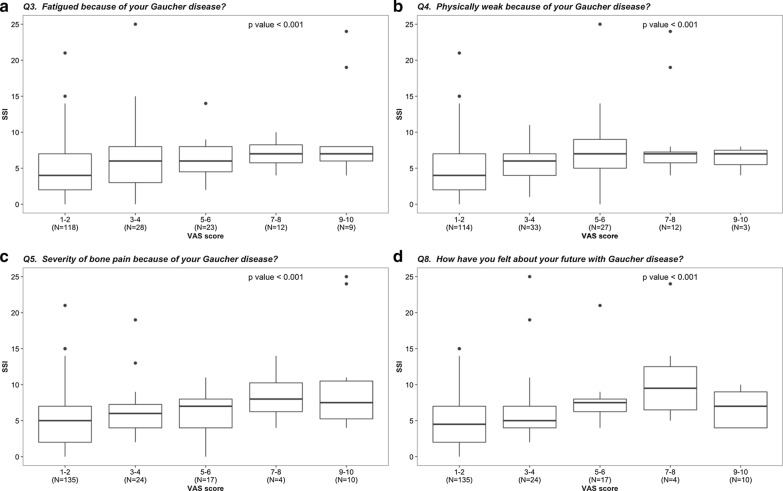
Significant association between the severity score index (SSI) and the responses on visual analogue scale (VAS) on the questions on **a** fatigue, **b** physical weakness, **c** severity of bone pain and **d** the way patients felt regarding their future

The responses to the eight VAS questions over the past week are presented in Table 3. The median (range) total VAS score was 1.6 (1–8.01). Patients receiving GD-specific therapy responded as less satisfied (higher mean and 95% CI for the mean) to all questions, except depression (question 6) (Table 3). The SSI at presentation was associated with fatigue, physical weakness, bone pain and the way patients felt regarding their future (Fig. 3). Sex age and lyso-Gb1 levels were not associated with responses to the eight VAS questions.

**Table 3:** Visual analogue scale (0–10) responses over the past week of patients with type I Gaucher disease

	All	Untreated group	Received GD-specific therapy	p value**
1. *Dependent* on others because of your Gaucher disease?	1.31 (1.2–1.4)	1.09 (0.9–1.28)	1.45 (1.23–1.68)	0.001
2. *Visibly big or swollen abdomen* because of your Gaucher disease?	2.05 (1.8–2.3)	1.70 (1.29–2.12)	2.18 (1.85–2.52)	0.06
3. *Fatigued* because of your Gaucher disease?	2.88 (2.5–3.2)	1.69 (1.37–2.00)	3.59 (3.09–4.08)	< 0.001
4. *Physically weak* because of your Gaucher disease?	2.73 (2.4–3.1)	1.70 (1.38–2.03)	3.39 (2.95–3.83)	< 0.001
5. *Severity of bone pain* because of your Gaucher disease?	2.40 (2.1–2.7)	1.57 (1.26–1.89)	2.88 (2.41–3.36)	< 0.001
6. *Depressed* because of your Gaucher disease?	1.62 (1.4–1.8)	1.35 (1.14–1.56)	1.80 (1.48–2.12)	0.18
7.* Worried* because of your Gaucher disease?	2.01 (1.7–2.3)	1.39 (1.17–1.60)	2.39 (1.99–2.78)	0.001
8. How have you felt about your *future* with Gaucher disease?	2.47 (2.2–2.8)	1.57 (1.28–1.87)	2.98 (2.55–3.41)	< 0.001

^*^Mean (95% confidence interval for the mean), ** Non-parametric independent samples Mann–Whitney test

The last VAS question reflects general satisfaction with the Gaucher medical treatment over the previous month. The mean (95% CI for the mean) of the entire cohort was 2.1 (1.8–2.4). The question was less relevant for the untreated cohort and was not associated with age, sex, SSI at presentation and recent lyso-Gb1 levels.

## Discussion

Our study is the first to show the PROM of a large cohort of patients with GD1 who were followed up regularly in a Gaucher Unit. Most patients reported that having GD did not restrict their education, job, intimate relations, hobbies or leisure activities and were not concerned about being an emotional burden to others because of their GD. Similarly, most patients' total VAS scores were low (median 1.6 from a maximum of 10). Still, a substantial group of patients reported that the GD affected their HRQoL, and future research is needed to find ways to improve their outcome. The lack of correlation between PROM and lyso-Gb1 reflects the importance of subjective PROM over an objective measurement of disease status.

The large cohort in our study enabled us to find predictors for the different PROM. The strongest predictor for good PROM was patients being untreated. This finding most probably results from the fact that the patients we choose not to treat are asymptomatic or mildly affected [6, 14], and thus their HRQoL is good. More restricted HRQoL among treated patients is most probably related to the baseline severity of the disease. Some of the patients had already suffered from irreversible complications, particularly skeletal, that could not be expected to improve significantly by treatment. Nevertheless, one cannot exclude the possibility that the therapy itself, particularly bi-weekly intravenous ERT, is the more unsatisfactory outcome. The sample size and the variety of treatments do not allow us to compare the effect of oral SRT with intravenous ERT on PROM [15].

An increased risk of the development of Parkinson disease exists in patients with GD and carriers of mutations in *GBA1*. There is considerable clinical variation; some patients have early-onset and prominent cognitive changes while others have a later onset and slower course [16–18]. The risk of Parkinson disease is increased both in patients who are c.1226A>G (N370S) homozygote and compound heterozygotes [19]; thus, it is not clear why patients with milder GD phenotype (that was found to be associated with c.1226A>G (N370S) homozygosity) were less concerned about the risk of Parkinson disease. Whether it is a lack of knowledge, misunderstanding, or the wrong perception of being healthy requires evaluation in future studies.

The only predictor of concern for the risk of cancer was female sex, as has also been shown in the general population [20]. Interestingly, although patients with GD have a somewhat increased risk of cancer, mainly multiple myeloma, almost two-thirds reported a lack of concern about the risk of cancer [21–24]. The risk of multiple myeloma and hepatocellular carcinoma (the second cancer type associated with GD) may decrease with the introduction of GD-specific therapy [25], and with the lower rate of splenectomy and hepatic fibrosis [26]. It has been recommended that all adult patients would be assessed for signs and symptoms of cancer as part of their routine follow-up visits [14].

Improving patient general wellbeing, mainly by reducing fatigue and restoring physical function, are included in the most recent consensus management goals of patients with GD [9]. Nevertheless, patients with higher SSI at presentation, although treated with GD-specific therapy, reported a relatively high score (median > 3) in both the fatigue and physical activity questions and also reported dissatisfaction with their current Gaucher medical treatment. The cause of ongoing, significant fatigue in patients with GD is not clear [27]. As endoplasmic reticulum (ER) stress could be an underlying mechanism [28, 29], pharmacological chaperones may have a role for reducing fatigue, and this may be uncovered by present and future studies [30]. The inflammatory changes that are not related to ER stress, and are not derived by inflammatory cytokines excreted by activated macrophages (the Gaucher cells) may require innovative therapeutic modalities, as has been suggested by several investigators [28]. One potential target might be the complement system, specifically the C5a/C5aR1 axis in GL1-specific autoantibody formation. Another one might be progranulin, as the low levels, commonly found among patients with GD, do not change with ERT and might induce some of the disease features [31].

Our study supports the advantage of electronic and mobile PROM surveys for simple quantitation of individual symptom items and aggregated scales, standardization, and longitudinal tracking of patient surveys for trend analysis over time [32]. Online PROM surveys were shown to achieve a significantly higher response rate, 40%–50%, compared to paper-based versions [33]. The use of mobile devices to communicate with the patients further simplified our study and increased the response rate [34]. The feasibility of pairing mobile devices with wearable technologies that can monitor surrogates of disease activity/severity could further improve our patients' management by highlighting personal problems that are not captured by the traditional parameters of physical signs, laboratory parameters, and imaging findings [31].

The study is limited by the fact that only 47% of adult patients with GD1 responded to the mobile PROM survey. We believe that not answering the survey was a combination of a lack of access to resources and engagement. Unfortunately, since we could include clinical data of only those consented, we could not compare those that answered to those that did not answer. Still, we were able to show that the age, sex, SSI, and homozygosity to the c.1226A>G (N370S) mutation of those that responded (study cohort) were similar to the entire SZMC database. A second limitation may relate to the fact that some of the questions are not specific to GD and may reflect sex and age group differences, as seen for the question on concern about cancer and effect on fun, hobbies, and leisure activities (Figs. 1a, 2).

## Conclusion

As is already known in other diseases, disease-specific PROMs are an important subjective tool for patient's evaluation and should be complementary to the objective assessment. Our study confirms that asymptomatic or mildly affected untreated patients with GD1 have good functional status and HRQoL, supporting our practice that not all patients with GD1 require specific therapy. Despite many years of available effective treatment for GD1, patients followed in our GD center report measures that may affect their functional status and HRQoL. Further work is needed to find effective interventions for such undesired measures. Electronic validated GD1-PROM, possibly via user-friendly GD-specific mobile applications, should be included among the outcome measures in clinical practice and all future prospective studies for GD.

## Data Availability

The datasets during and/or analysed during the current study available from the corresponding author on reasonable request.

## Abbreviations

ERT: Enzyme replacement therapy
GD: Gaucher disease
GD1: Gaucher disease type 1
HRQOL: Health related quality of life
PROM: Patient-reported outcome measures
SSI: Severity score index
SZMC: Shaare Zedek Medical Center

## References

[1] Slade A, Isa F, Kyte D, Pankhurst T, Kerecuk L, Ferguson J, Lipkin G, Calvert M (2018). Patient reported outcome measures in rare diseases: a narrative review. Orphanet J Rare Dis.

[2] Snyder CF, Aaronson NK (2009). Use of patient-reported outcomes in clinical practice. Lancet.

[3] Black N (2013). Patient reported outcome measures could help transform healthcare. Br Med J.

[4] Zimran A, Belmatoug N, Bembi B, Deegan P, Elstein D, Fernandez-Sasso D, Giraldo P, Goker-Alpan O, Lau H, Lukina E (2018). Demographics and patient characteristics of 1209 patients with Gaucher disease: descriptive analysis from the Gaucher Outcome Survey (GOS). Am J Hematol.

[5] Zimran A, Elstein D, Lichtman M, Beutler E, Kipps TJ, Seligsohn U, Prchal J (2016). Gaucher disease and related lysosomal storage diseases. Williams' Hematology.

[6] Dinur T, Zimran A, Becker-Cohen M, Arkadir D, Cozma C, Hovakimyan M, Oppermann S, Demuth L, Rolfs A, Revel-Vilk S (2019). Long Term follow-up of 103 untreated adult patients with type 1 gaucher disease. J Clin Med.

[7] Gary SE, Ryan E, Steward AM, Sidransky E (2018). Recent advances in the diagnosis and management of Gaucher disease. Expert Rev Endocrinol Metab.

[8] Beutler E (1994). Economic malpractice in the treatment of Gaucher's disease. Am J Med.

[9] Biegstraaten M, Cox TM, Belmatoug N, Berger MG, Collin-Histed T, Vom Dahl S, Di Rocco M, Fraga C, Giona F, Giraldo P (2018). Management goals for type 1 Gaucher disease: an expert consensus document from the European working group on Gaucher disease. Blood Cells Mol Dis.

[10] Johnston BC, Miller PA, Agarwal A, Mulla S, Khokhar R, De Oliveira K, Hitchcock CL, Sadeghirad B, Mohiuddin M, Sekercioglu N (2016). Limited responsiveness related to the minimal important difference of patient-reported outcomes in rare diseases. J Clin Epidemiol.

[11] Elstein D, Klemen M, Panter C, Bonner N, Johnson C, Zimran A (2019). Gaucher disease (GD)-specific patient-reported outcome (PRO) measures for clinical monitoring and for clinical trials. Mol Genet Metab.

[12] Generational breakdown: info about all of the generations. https://genhq.com/FAQ-info-about-generations/.

[13] Zimran A, Sorge J, Gross E, Kubitz M, West C, Beutler E (1989). Prediction of severity of Gaucher's disease by identification of mutations at DNA level. Lancet.

[14] Revel-Vilk S, Szer J, Mehta A, Zimran A (2018). How we manage Gaucher Disease in the era of choices. Br J Haematol.

[15] Wagner VF, Northrup H, Hashmi SS, Nguyen JM, Koenig MK, Davis JM (2018). Attitudes of Individuals with Gaucher Disease toward Substrate Reduction Therapies. J Genet Couns.

[16] Lopez G, Kim J, Wiggs E, Cintron D, Groden C, Tayebi N, Mistry PK, Pastores GM, Zimran A, Goker-Alpan O (2016). Clinical course and prognosis in patients with Gaucher disease and parkinsonism. Neurol Genet.

[17] Aflaki E, Westbroek W, Sidransky E (2017). The Complicated relationship between Gaucher disease and parkinsonism: insights from a rare disease. Neuron.

[18] Alcalay RN, Dinur T, Quinn T, Sakanaka K, Levy O, Waters C, Fahn S, Dorovski T, Chung WK, Pauciulo M (2014). Comparison of Parkinson risk in Ashkenazi Jewish patients with Gaucher disease and GBA heterozygotes. JAMA Neurol.

[19] Chetrit EB, Alcalay RN, Steiner-Birmanns B, Altarescu G, Phillips M, Elstein D, Zimran A (2013). Phenotype in patients with Gaucher disease and Parkinson disease. Blood Cells Mol Dis.

[20] McQueen A, Vernon SW, Meissner HI, Rakowski W (2008). Risk perceptions and worry about cancer: does gender make a difference?. J Health Commun.

[21] Zimran A, Liphshitz I, Barchana M, Abrahamov A, Elstein D (2005). Incidence of malignancies among patients with type I Gaucher disease from a single referral clinic. Blood Cells Mol Dis.

[22] Rosenbloom BE, Weinreb NJ, Zimran A, Kacena KA, Charrow J, Ward E (2005). Gaucher disease and cancer incidence: a study from the Gaucher Registry. Blood.

[23] Arends M, van Dussen L, Biegstraaten M, Hollak CE (2013). Malignancies and monoclonal gammopathy in Gaucher disease; a systematic review of the literature. Br J Haematol.

[24] Weinreb NJ, Mistry PK, Rosenbloom BE, Dhodapkar MV (2018). MGUS, lymphoplasmacytic malignancies, and Gaucher disease: the significance of the clinical association. Blood.

[25] Martinez-Redondo C, Ortuno FJ, Lozano ML, Jerez A, del Mar OM, Giraldo P, Vicente V (2009). IgM monoclonal component associated with type I Gaucher disease resolved after enzyme replacement therapy: a case report. J Inherit Metab Dis.

[26] Regenboog M, van Dussen L, Verheij J, Weinreb NJ, Santosa D, Vom Dahl S, Haussinger D, Muller MN, Canbay A, Rigoldi M (2018). Hepatocellular carcinoma in Gaucher disease: an international case series. J Inherit Metab Dis.

[27] Zion YC, Pappadopulos E, Wajnrajch M, Rosenbaum H (2016). Rethinking fatigue in Gaucher disease. Orphanet J Rare Dis.

[28] Pandey MK, Grabowski GA, Kohl J (2018). An unexpected player in Gaucher disease: the multiple roles of complement in disease development. Semin Immunol.

[29] Maor G, Rencus-Lazar S, Filocamo M, Steller H, Segal D, Horowitz M (2013). Unfolded protein response in Gaucher disease: from human to Drosophila. Orphanet J Rare Dis.

[30] Babajani G, Tropak MB, Mahuran DJ, Kermode AR (2012). Pharmacological chaperones facilitate the post-ER transport of recombinant N370S mutant beta-glucocerebrosidase in plant cells: evidence that N370S is a folding mutant. Mol Genet Metab.

[31] Jian J, Zhao S, Tian QY, Liu H, Zhao Y, Chen WC, Grunig G, Torres PA, Wang BC, Zeng B (2016). Association between Progranulin and Gaucher Disease. EBioMedicine.

[32] Schwartzberg L (2016). Electronic patient-reported outcomes: the time is ripe for integration into patient care and clinical research. Am Soc Clin Oncol Educ Book.

[33] Horevoorts NJ, Vissers PA, Mols F, Thong MS, van de Poll-Franse LV (2015). Response rates for patient-reported outcomes using web-based versus paper questionnaires: comparison of two invitational methods in older colorectal cancer patients. J Med Internet Res.

[34] Bae WK, Kwon J, Lee HW, Lee SC, Song EK, Shim H, Ryu KH, Song J, Seo S, Yang Y (2018). Feasibility and accessibility of electronic patient-reported outcome measures using a smartphone during routine chemotherapy: a pilot study. Support Care Cancer.

